# Assessing the Morphological and Behavioral Toxicity of Catechol Using Larval Zebrafish

**DOI:** 10.3390/ijms23147985

**Published:** 2022-07-20

**Authors:** Michael G. Morash, Kelly H. Soanes, John C. Achenbach, Lee D. Ellis

**Affiliations:** 1 Aquatic Crop and Resource Development, National Research Council of Canada, Halifax, NS B3H 3Z1, Canada; john.achenbach@nrc-cnrc.gc.ca (J.C.A.); lee.ellis@nrc-cnrc.gc.ca (L.D.E.); 2 Aquatic Crop and Resource Development, National Research Council of Canada, Saskatoon, SK S7N 0W9, Canada; kelly.soanes@nrc-cnrc.gc.ca

**Keywords:** catechol, zebrafish, toxicity, gene expression, melanocytes, behavior

## Abstract

Catechol is a ubiquitous chemical used in the manufacturing of fragrances, pharmaceuticals and flavorants. Environmental exposure occurs in a variety of ways through industrial processes, during pyrolysis and in effluent, yet despite its prevalence, there is limited information regarding its toxicity. While the genotoxicity and gastric carcinogenicity of catechol have been described in depth, toxicological studies have potentially overlooked a number of other effects relevant to humans. Here, we have made use of a general and behavioral larval zebrafish toxicity assay to describe previously unknown catechol-based toxicological phenomena. Behavioral testing revealed catechol-induced hypoactivity at concentrations an order of magnitude lower than observable endpoints. Catechol exposure also resulted in punctate melanocytes with concomitant decreases in the expression of pigment production and regulation markers *mitfa*, *mc1r* and *tyr*. Because catechol is converted into a number of toxic metabolites by tyrosinase, an enzyme found almost exclusively in melanocytes, an evaluation of the effects of catechol on these cells is critical to evaluating the safety of this chemical. This work provides insights into the toxic nature of catechol and highlights the benefits of the zebrafish larval testing platform in being able to dissect multiple aspects of toxicity with one model.

## 1. Introduction

Catechol (pyrocatechol; 1,2-dihydroxybenzene) is used primarily in the synthesis of insecticides such as Propoxur and Carbofuran, in the production of artificial flavors such as Vanillin, and as a precursor to a variety of pharmaceuticals [1]. Human exposure to catechol occurs from numerous sources, including during its manufacturing [2], as a contaminant of effluents from a variety of industries (e.g., textiles, pulp and paper, petrochemicals) [3], and from the pyrolysis of lignin [4], tobacco [5,6] and cannabis [7]. Catechol is also commercially available as a photo developer, in specialty inks and as a dying agent [2]. Additionally, although catechol is rarely used directly in cosmetic agents due to its toxicity, catechol moieties are found in skin-whitening products [8] and hair dyes [9].

Oral administration studies have demonstrated carcinogenic activity of catechol in rodents, but due to limited exposure data catechol remains classified as only a possible human carcinogen [2]. Catechol’s genotoxic actions are mediated primarily through its ability to induce adduct formation [10,11,12,13] and DNA strand breaks [11]. Despite its ubiquity and demonstrated carcinogenic activity, aside from the calculation of LD_50_ values, few studies have focused on the holistic, extra-carcinogenic toxicological effects on whole organisms. There then remain limited data regarding its toxicity.

The value and predictability of using zebrafish larvae for toxicity testing are widely accepted, and a number of standardized tests have been developed. Specifically, the fish embryo toxicity assay (FET) has been recognized by the Organization for Economic Co-operation and Development (OECD) as a predictive model for environmental risk assessment [14]. To complement these tests, previous work from our lab has developed a standardized model of general and behavioral toxicity (GBT) [15]. The GBT assay evaluates the toxic effects of a compound at the larval stage of development, which is considered the point at which body patterning has been established. Thus, any toxic effect that is produced is less likely to be the result of teratogenicity and is more likely to be predictive of the patterns of toxicity found in adults. Additionally, the GBT assay includes a behavioral component that has proven to be more sensitive than phenotypic toxicity assessment, most notably when chemicals have neurotoxic activity [16].

The current study assessed the effectiveness and predictability of the GBT assay for studying the toxic effects of catechol on zebrafish larvae. In addition to the initial lethality testing, the GBT assay allowed us to demonstrate sub-phenotypic effects on behavior, quantify altered melanocyte morphology and analyze gene expression changes. This work provides new insights into the toxicity profile of catechol and further highlights the usefulness of the GBT assay for assessing compounds with multiple mechanisms of toxicity.

## 2. Results

### 2.1. Determination of LC_50_ Values for Catechol on Zebrafish Larvae

**Figure 1:**
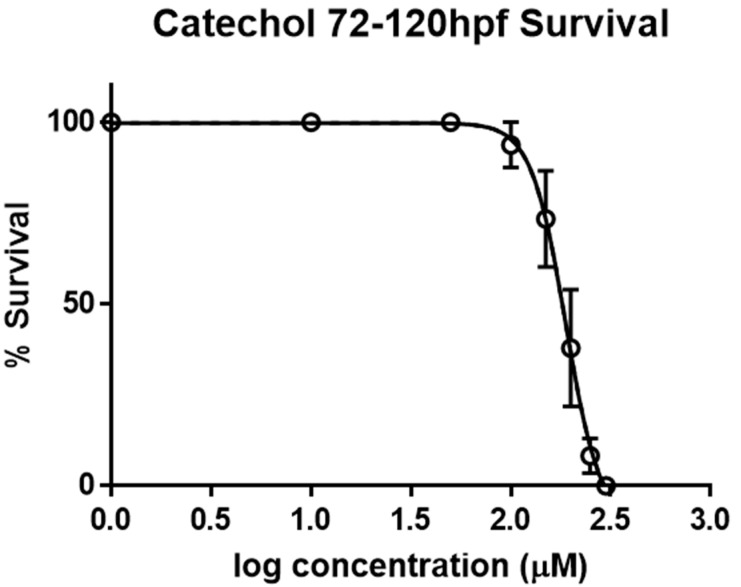
Determination of LC_50_ values for catechol exposure against 72–120 hpf zebrafish larvae. In total, 72 hpf zebrafish embryos were exposed to catechol in HEPES buffered E3 (HE3) containing 0.5% DMSO for 48 h. Percent survival is calculated as 100-(number of dead embryos/number of total embryos × 100) and then plotted as four-parameter variable slope curve.

The initial assessment of the toxicity of catechol on zebrafish larvae made use of previously defined models of general and behavioral toxicity (GBT) [15]. Briefly, larvae were exposed to catechol from 72–120 h post fertilization (hpf) and were scored for lethality. The LC_50_ for catechol at 120 hpf was 188.8 µM (C.I. 159–224 µM, Figure 1). The predominant observable phenotype was a decrease in visible pigmentation (see below).

### 2.2. Behavioral Effects of Catechol

**Figure 2:**
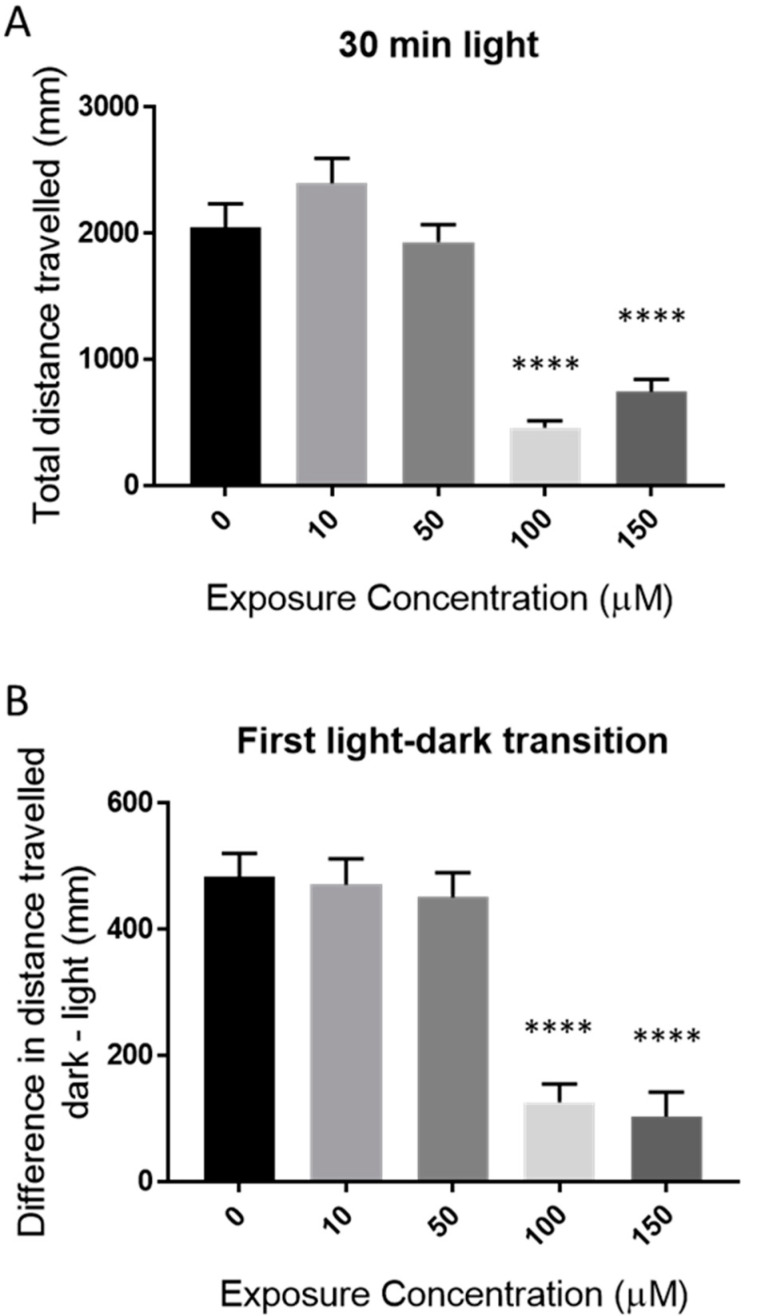
Behavioral effects of catechol on 72 hpf zebrafish larvae. Zebrafish larvae were exposed to a range of catechol concentrations for 48 h. Their activity was measured as total distance traveled over 30 min (**A**) and the first light–dark transition (**B**). Asterisks represent differences between concentrations, **** *p* < 0.0001.

Catechol exposure resulted in decreased locomotor activity during the initial baseline of 30 min of the behavioral protocol at 100 µM and 150 µM (Figure 2A). There was a similar decrease in the first light–dark transition (startle response) (Figure 2B).

### 2.3. Catechol Affects Pigmentation in Zebrafish Larvae

**Figure 3:**
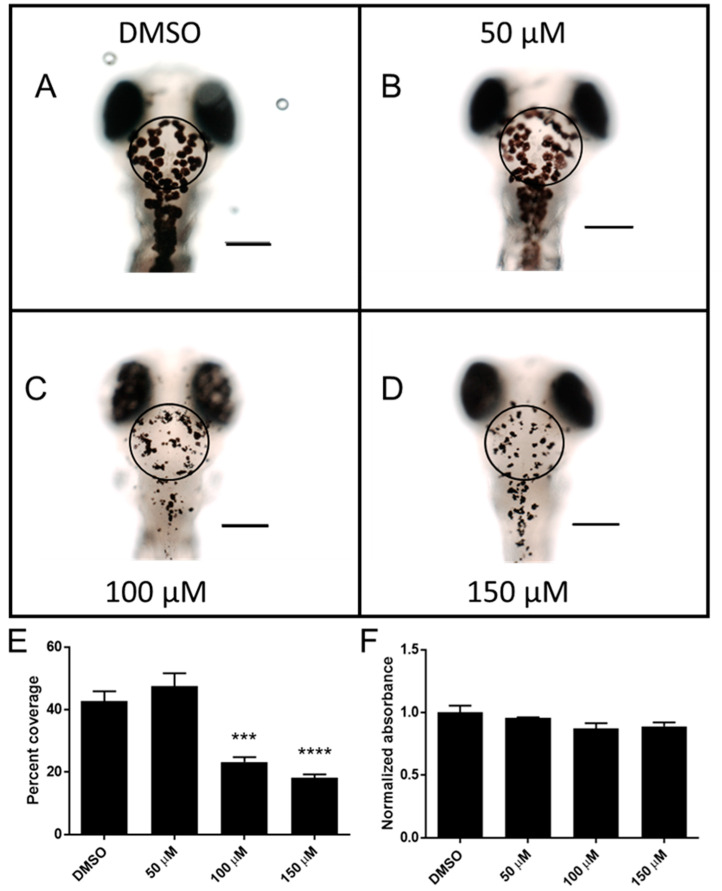
Phenotypic changes due to catechol treatment. In total, 72 hpf larvae were exposed to increasing concentrations of catechol for 48 h under dark conditions prior to fixation. Representative images of each treatment are shown in panels (**A**–**D**) ((**A**)—DMSO, (**B**)—50 µM, (**C**)—100 µM, (**D**)—150 µM). The areas used to calculate percent coverage are shown (circles), scale bars—0.2 mm. Percent coverage of melanocyte compared to total area was calculated for 10–14 embryos and is shown in panel (**E**). Melanin quantification was also performed on similarly treated, unfixed embryos (panel (**F**)). *** *p* < 0.001, **** *p* < 0.0001 compared to DMSO, one-way ANOVA, Bonferroni’s post hoc test.

The most significant observable effect that was produced by sub-lethal exposure to catechol was an apparent alteration in melanocyte size. Initially, this change was difficult to quantify due to the punctate nature of the melanocytes. When larvae are placed in a dark environment, their normal camouflage response produces a dispersion of melanocytes [17]. In order to promote melanocyte dispersion, larvae were placed in the dark during exposure to catechol. Microscopic examination of the catechol-exposed larvae revealed that at 100 µM and 150 µM, catechol disrupted normal melanocyte appearance (Figure 3C,D). There were also several regions with extremely small deposits of melanin. Melanocytes appeared punctate, less dendritic and irregularly dispersed. Image analysis revealed that both 100 µM and 150 µM-treated embryos had significantly less melanocyte coverage than the DMSO controls (DMSO 23.3 ± 1.6% and 18.2 ± 1.1% vs. 42.6 ± 3.2% vs. respectively, Figure 3E). The lowest dose of catechol (50 µM) did not significantly change melanocyte coverage. Exposure to catechol did not produce a significant decrease in total melanin in treated larvae after 48 h exposure (Figure 3F). Melanin levels did trend towards a decrease, but this was not statistically significant.

### 2.4. Catechol Exposure Affects the Expression of Several Melanocyte-Related Genes

**Figure 4:**
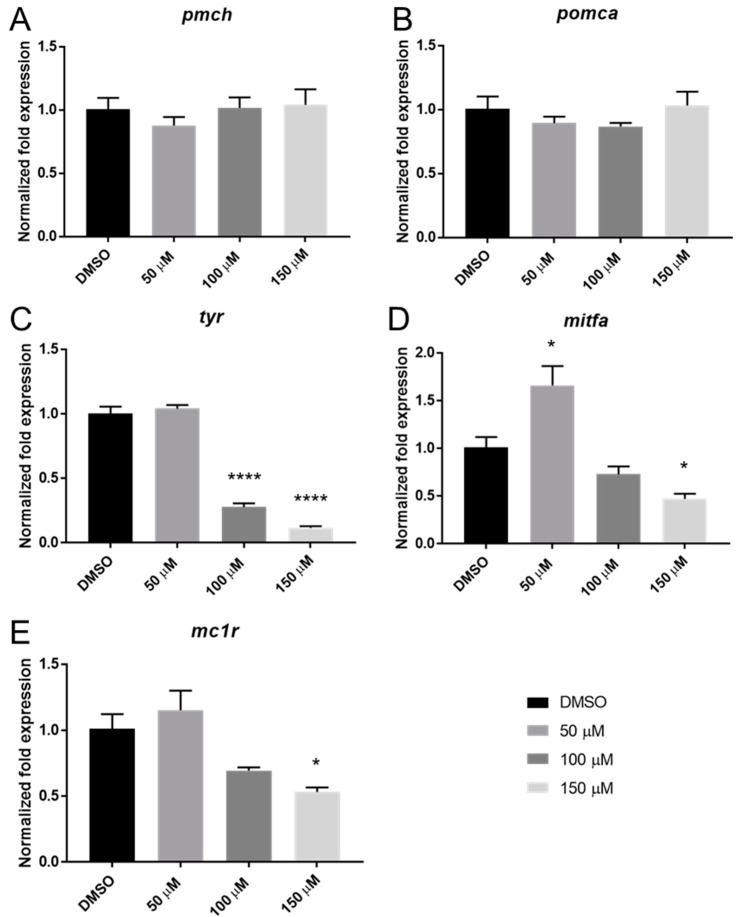
Effects of 72–120 hpf exposure to catechol on melanocyte-related gene expression. In total, 72 hpf embryos were treated for 48 h in DMSO or catechol, and then the gene expression of *pmch* (**A**), *pomca* (**B**), *tyr* (**C**), *mitfa* (**D**) and *mc1r* (**E**) were analyzed by qPCR at 120 hpf. * *p* < 0.05; **** *p* < 0.0001. One-way ANOVA with Bonferroni’s post hoc test. For the purposes of clarity, statistical significance is only shown when a difference is significant compared to DMSO control.

To investigate the mechanism of the catechol-based pigmentation effects, the expression of several melanocyte-related genes was measured (Figure 4). *tyr* expression (Figure 4C) was decreased at both 100 µM and 150 µM (*p* < 0.0001). At 150 µM, catechol induced a significant decrease in the expression of the pigment-related genes *mitfa* (*p* < 0.05) and *mc1r* (*p* < 0.05) (Figure 4D,E respectively). Interestingly, 50 µM catechol caused a significant increase in *mitfa* expression (Figure 4D).

## 3. Discussion

Catechol is a ubiquitous phenolic compound used in a range of industries, including pharmaceuticals and fragrance manufacturing, and as a precursor for a variety of chemicals. Human environmental exposure occurs through a variety of methods, including as the byproduct of pulp and paper production, release from wildfires, and due to its prominence in tobacco and cannabis smoke.

Our lethality data are in line with previously published results of ~100 µM using a standard OECD assay [4]. There is little description of behavioral toxicity of catechol in the literature, although it is known to produce similar neurotoxicity to phenol in human poisoning [18]. Catechol exposure led to a significant reduction of larval locomotor activity well below the calculated LC_50_ value. It has previously been suggested that such effects can be the result of changes in neuromuscular function or, as is more likely in this case, the result of neurotoxicity [19,20]. More generally, behavioral analysis of larvae has been used as a high throughput method for studying the effects of many pharmacological agents [21]. The results described in this work suggest catechol may have neurotoxic effects, and further studies are warranted.

At sub-lethal concentrations, the most overt phenotype observed following catechol exposure was a change in melanocyte distribution and intensity. Zebrafish larvae typically display a visual background adaptation that results in the aggregation or dispersion of melanosomes in response to ambient light or dark, respectively [17,22]. In order to maximize melanocyte coverage of larvae, they were treated with catechol in a dark environment. Catechol is a substrate for tyrosinase, an enzyme found in melanocytes responsible for two steps in melanin production; the ortho-hydroxylation of monophenols and the oxidation of those diphenols into ortho-quinones [23]. Ortho-quinones are highly reactive, causing cell damage by binding free thiol groups and through the generation of reactive oxygen species [24]. Tyrosinase expression is unique to melanocytes and their progenitor cells [25], and given that the toxicity of catechol is greatly enhanced by tyrosinase activity, melanocytes represent an ideal cell type for studying its toxicity. Indeed zebrafish melanocytes have been used to elucidate the biological mechanisms of a number of human conditions, including melanoma, vitiligo and albinism [26]. Given the mechanism of catechol toxicity, a potential role in melanoma should be investigated.

The level of pigmentation in zebrafish is controlled both by the autonomic nervous system, which allows almost immediate control of pigmentation used in social signaling (mating, aggression) and hormonally through a number of factors, including MITFA [27]. In the current study, it was found that catechol decreases the expression of *mitfa*, and 2 *mitfa* controlled genes, *mc1r* and *tyr*. MITFA is required at multiple steps of melanocyte development, and *mitfa* disruption has been shown to result in melanocytes that lose dendricity, similar to those observed herein [28]. The loss of *mitfa* expression after catechol exposure may lead to an MC1R receptor decrease, which could attenuate α-melanocortin stimulating (αMSH) signaling and allow for only promelanin concentrating hormone (PMCH) signaling. The antagonistic relationship between αMSH, which promotes melanocyte expansion, and a second hormone, PMCH, which promotes aggregation, are largely responsible for melanocyte behavior [29]. It is tempting to speculate that catechol’s effect on melanocyte aggregation is mediated through catecholamine-receptor binding. Several catecholamines, including noradrenaline, cause melanosome aggregation by inhibiting cAMP production via αMSH signaling through the MC1R receptor [30,31]. Furthermore, dissection of the binding mechanics of the B2 adrenergic receptor has demonstrated that catechol itself is an agonist of this receptor [32]. Thus, the aggregation of melanosomes seen upon catechol exposure may be caused by inhibition of adenylate cyclase through catechol signaling via the adrenergic receptors. Consistent with the idea that melanosome aggregation was responsible for the decreased size of the melanocytes, there was no statistically significant decrease in melanin content, suggesting that the changes in pigmentation are not due to inhibition of melanin synthesis, despite the decrease in *tyr* expression. Additionally, by 72 hpf, melanocyte numbers have reached a maximum, so the lack of pigment in larvae treated at 72 hpf is probably not due to a decrease in melanocyte proliferation [33]. The decreased tyrosinase expression may, however, eventually lead to a decrease in melanin levels if the experimental window were increased.

This paper describes the toxicity of catechol on zebrafish and describes its effects on survival, behavior, melanocyte morphology and selected gene expression. Importantly, this work demonstrates the use of the standardized GBT assay to test multiple effects of a known toxicant. By taking advantage of multiple aspects of toxicity, the GBT assay offers several starting points with which to begin to identify the molecular mechanisms that are leading to the generation of specific toxic phenotypes.

## 4. Materials and Methods

### 4.1. Zebrafish Husbandry and Embryo Collection

A hybrid AB/Tubingen zebrafish line (created in house) was maintained at 28 °C on a 14 h light–10 h dark cycle according to standard culture conditions [34] in a ZebTEC housing system (Tecniplast USA, Easton, PA, USA), and in accordance with Canadian Council for Animal Care guidelines. Age-matched embryos were sorted for fertilization at roughly 4 h post-fertilization (hpf) and were transferred to Pentair Aquatic Ecosystem (Apopka, FL, USA) nursery baskets (maximum 300 embryos per basket) residing in a 3 L tank on the ZebTEC system and raised until 72 hpf at 28.5 ± 0.5 °C on a 14:10 light–dark cycle. All adult zebrafish husbandry and breeding were in accordance with the Canadian Council of Animal Care (CCAC) guidelines.

### 4.2. Chemicals

Catechol (CAS# 120-80-9, Cat# C-9510, purity ≥ 99%) (Sigma-Aldrich, Oakville, ON, Canada) was dissolved in dimethyl sulfoxide (DMSO) (Cat# D8418, purity ≥ 99%) (Sigma-Aldrich, Oakville, ON, Canada) at a stock concentration of 200 mM solution and stored at 4 °C until used.

### 4.3. Chemical Larval Exposure

At 72 h post fertilization (hpf), embryos were transferred in 150 µL of E3 (5 mM NaCl, 0.17 mM KCL, 0.33 mM CaCl_2_∙2H_2_O, 0.33 mM MgSO_4_) media buffered with 10 mM HEPES pH 7.2, (HE3) into 96-well plates, where they received 150 µL of a 2X dilution of catechol normalized in 1% DMSO (*v/v*), for a final concentration of 0.5% DMSO (*v/v*), and then covered with sealing film, and placed in a light–dark incubator (14 h light: 10 h dark). 0.5% DMSO (*v/v*) in HE3 was used as a carrier control, and catechol solutions were not replaced during exposures.

### 4.4. Behavioral and Toxicity Testing

Prior to 120 hpf phenotype scoring, the plates were placed into a Zebrabox, and their activity was monitored using the Viewpoint video tracking system and software (Viewpoint Life Sciences Inc., Montréal, QC, Canada). The plate temperature was maintained in the Zebrabox chamber at 28 °C by partial immersion in a re-circulating water bath. All experiments consisted of 30 min light followed by three 10 min cycles containing a dark and light phase (5 min each). The total locomotor activity as measured by total distance traveled for the first 30 min and during the 1st and second 2nd light–dark transitions were analyzed. Replicate experiments were run on two or three separate days (*n* = 12/day) and pooled for subsequent analysis (*n* = 24–36). Embryos were assessed for indications of toxicity or lethality at 120 hpf immediately following behavioral tracking. All phenotypically affected or dead larvae were removed from analysis. Treatment groups were compared to controls, and statistical significance was determined by one-way ANOVA, followed by Dunnett’s post hoc test (*p* < 0.05) using recommended parametric assumptions of variance and distribution (Brown–Forsythe and Bartlett’s tests). Lethality data were transformed as log (concentration), followed by nonlinear regression (variable slope–four parameters) to calculate Lethal Concentration 50% (LC_50_) values using Graphpad Prism 5 (GraphPad Software, San Diego, CA, USA).

### 4.5. Melanin Quantification

In total, 72 hpf embryos (30 per group) were treated with 50, 100 or 150 µM catechol exactly as described above, except larvae were incubated in the dark. A 0.5% DMSO (*v/v*) carrier control treatment was also performed. At 120 hpf, embryos from three wells were pooled, and five were randomly removed to 4% paraformaldehyde (*w/v*) (PFA, Sigma-Aldrich, Oakville, ON, Canada) and stored at 4 °C for up to one week for imaging (see below). The remaining embryos were transferred to ice, and the media were removed. Melanin was quantified similarly to the method described by Lin et al. [35]. Briefly, embryos were lysed by pipetting in 1 mL of lysis buffer (20 mM sodium phosphate buffer (pH 6.8), 1 mM PMSF and 1% Triton X-100), containing EDTA-free protease inhibitor (Roche, Montreal, QC, Canada). Supernatants were centrifuged for 10 min, 14,000× *g* at room temperature (RT), and all buffer removed. Pellets were resuspended in 350 µL of solubilizing buffer (1 N NaOH, 20% DMSO) and heated to 95 °C for 30 min with periodic shaking by hand. Once the melanin had dissolved, samples were centrifuged at 14,000× *g* for 1 min at RT to remove any insoluble debris, and 100 µL pipetted into triplicate wells of a 96-well plate, and the absorbance at 490 nm was read. The data from three separate treatment groups were then averaged and normalized to the DMSO carrier control.

The PFA-fixed embryos were used for melanocyte area coverage calculations. Fixative was removed and replaced with HE3 media, and the five embryos from a single tube were transferred to a depression slide, the media removed and replaced with 250 µL of 1.5% methylcellulose for orientation and imaging. Embryos were imaged using a Nikon AZ-100 stereomicroscope, with an AZ Plan Apo 4× lens and 4× zoom. All images were processed using NIS Elements BR 2.30 software (Nikon Instruments Inc. Melville, NY). For each image, a defined circular region of interest (ROI) was placed over the dorsal melanocytes just behind the eyes (see Figure 3 for placement examples). The intensity threshold of the image was then adjusted when required to remove background (non-melanocyte) pixel contribution within the ROI, and if necessary, the ROI was shifted slightly to avoid pixel contribution from the eyes. The area fraction was then automatically calculated as the binary area/ROI area × 100. Images (10–14 per concentration) were used for calculating percent coverage.

### 4.6. QPCR Chemical Challenge

Experiments were performed as previously described [15]. Briefly, 72 hpf larvae were transferred to 6-well plates at a density of 10 per well in E3 media. The media was replaced with HE3 media and then spiked with the appropriate volume of catechol or DMSO to obtain the desired concentration in a total of 3 mL. Control wells were standardized to 0.5% DMSO (carrier control) and plates incubated under standard light–dark cycling. After 48 h, embryos were examined microscopically to remove any dead embryos, and three wells of either catechol-treated or DMSO-treated control larvae were pooled. Embryos were anesthetized on ice, frozen on dry ice, and stored at −80 °C until processing.

### 4.7. RNA Extraction and cDNA Synthesis

Embryos were thawed on ice, and RNA was isolated using a Total RNA Purification Kit (Norgen Biotek, Thorold, ON, Canada) following the manufacturer’s instructions for animal tissues with the following modifications: 30 larvae per tube were homogenized by pipetting in 500 µL of lysis buffer containing 1% β-mercaptoethanol (Sigma-Aldrich, Oakville, ON, Canada), and centrifuged for 5 min at RT, at 14,000× *g*. After ethanol addition, samples were centrifuged for 2 min, RT, 14,000× *g*. The optional on-column DNase I digestion was performed using RNase-free DNase I (Norgen Biotek, Thorold, ON, Canada). Finally, RNA was eluted in 30 µL of elution buffer. The concentration and purity of the RNA were then determined spectrophotometrically, and 1 µg of RNA was used to synthesize cDNA using Superscript III reverse transcriptase (Invitrogen, Burlington, ON, Canada) according to the manufacturer’s recommendation. Controls containing no reverse transcriptase were performed on all RNA samples. All cDNA was stored at −20 °C until use.

### 4.8. qPCR

All qPCR reactions were performed in 10 µL volumes in 384-well PCR plates using a Roche LightCycler 480 thermal cycler (Roche, Laval, QC, Canada) as described previously using previously published primer sets [15] and Mc1r (F-GCACATGTTCATCTTGGCCCATGT, R-AGGGTTTGTGGGACAGGTGAGAAT). Each reaction received 4 µL of cDNA (diluted 1/15 with deionized water (Sigma-Aldrich, Oakville, ON, Canada)) and 6 µL of enzyme/primer premix (5 µL of 2× KAPA SYBR FAST qPCR Master Mix (Sigma-Aldrich, Oakville, ON Canada), 0.25 µL of each 10 mM primer ), and 0.5 µL of deionized water (Sigma-Aldrich, Oakville, ON, Canada). The program consisted of 45 cycles of amplification (melt 95 °C, anneal 60 °C, extension 72 °C) followed by a melt curve. Three technical replicates were run for each primer pair and cDNA per plate, and three biological replicates were performed. Data were analyzed as follows: technical repeats were assessed (melt curve, C_t_), and C_t_ values were averaged. ∆∆C_t_ was calculated using the housekeeping gene (*ef-1α*). As a control, analysis was also performed using the *rpl13* transcript to validate the stability of the *ef-1α* transcript (not shown). Statistical analysis was performed on the *ef-1α* normalized values. Statistical significance was assessed using one-way ANOVA with a Dunnett’s post hoc test (*p* < 0.05) (Graphpad Software, San Diego, CA, USA) using recommended parametric assumptions of variance and distribution (Brown–Forsythe and Bartlett’s tests).

## Data Availability

Data available upon request.
